# Hexahedral mesh generation via constrained quadrilateralization

**DOI:** 10.1371/journal.pone.0177603

**Published:** 2017-05-18

**Authors:** Feifei Shang, Yangke Gan, Yufei Guo

**Affiliations:** Department of Mechanics and Engineering Science, College of Engineering, Peking University, Beijing, China; Consiglio Nazionale delle Ricerche, ITALY

## Abstract

Decomposing a volume into high-quality hexahedral cells is a challenging task in finite element simulations and computer graphics. Inspired by the use of a spatial twist continuum and frame field in previous hexahedral mesh generation methods, we present a method of hexahedral mesh generation via constrained quadrilateralization that combines a spatial twist continuum and frame fields. Given a volume represented by a tetrahedral mesh, surface quadrilateral mesh and frame field, we first extend the loop of the surface of a solid to a layer of hexahedral elements, then divide the solid into two smaller sub-solids by the layer, and finally handle them recursively until all of the sub-solids are empty. In our hexahedral mesh generation framework, we apply constrained quadrilateralization to extend the loop to a layer of hexahedral elements. The “divide-and-conquer” strategy used in this method is suitable for parallelization. This method can potentially lead to easier and more robust implementations that are more parallelizable and less dependent on heavy numerical libraries. The testing results show that the quality of the meshes generated by this method is similar to those produced by current state-of-the-art mesh generation methods.

## 1. Introduction

Hexahedral meshes (or more colloquially, hex meshes) have many applications in finite element simulations and computer graphics [1–5]. Hex mesh generation has been considered as the Holy Grail in the mesh generation community for many years [6–8], as such meshes can improve both speed and accuracy. A wide variety of methods have been proposed in the literature to solve the hexahedral mesh generation problem. These methods were divided by Owen[1] into three families: direct, indirect, and structured methods. With a direct approach, hexes are placed in the domain directly, without a preceding process of tetrahedral meshing. Grid-based[7, 9–11], skeleton-based[12–15], whisker weaving[16–20] and plastering[21–25] are four kinds of direct methods. In an indirect approach, the domain is first meshed with tetrahedrons. Various algorithms are then employed to convert the tetrahedrons into hexes. The disadvantage of these methods is that the quality of the resultant hex mesh can be very poor owing to the high valence nodes [26, 27]. A structured hex mesh is a mesh whose inner edge valence is only four. A famous structured method is mapping[28], by which a map from a given solid with six surfaces to a cuboid is constructed. The frame field-based method[8, 29–32]and the PolyCube[14, 33–37] method are two popular recent variations. Among all these methods, two attract our attention, namely, the whisker weaving method and the frame field-based method.

The whisker weaving method [16] is an enlightened method for hex mesh generation. It first builds the combinatorial dual of a mesh and constructs the primal mesh and its embedding only afterwards. The whisker weaving method was developed by Folwell and Mitchell [17] and Müller–Hannemann [18]. They have successfully converted a degenerated hex into a well-defined hex. Recently, Kawamyra *et al*. [19] improved the whisker weaving method by creating elements and nodes in 3D real space during the weaving process. It is possible to generate a hex mesh with fewer low-quality elements. Kremer[20] introduced several techniques to extend the scope of target shapes from the“almost convex” objects which Müller–Hannemann can only handle to concave geometry.

The frame field-based method is a popular hex meshing method that is often used in computer graphics. This method generally involves three steps: 1) constructing a 3D frame field, 2) computing a volumetric parameterization guided by this field, and 3) extracting a hex mesh to which the parameterization leads. Recently, Li *et al*. [38] proposed a typical example of the method. The key of their work is a novel frame field called the singularity-restricted field, which is obtained automatically. Jiang *et al*. [8] proposed a systematic treatment that removes some of the singularities that would lead to degenerating volumetric parameterization. Kowalskia *et al*. [32] proposed a novel algorithm to generate block-structures that are adapted to hexahedral mesh generation using a frame field. Instead of performing a parameterization algorithm, a block structure is built on the singularity graph of the frame field.

The whisker weaving method is an advancing front method and can maintain high quality of the boundary mesh. However, it advances the front only by local optimum and lacks global consideration. The frame field-based method has recently become very popular. It generates volumetric parameterization using the frame field. This method always depends heavily on numerical libraries. It is difficult to generate a suitable parameterization, and treatments to revise the parameterization are required in many frame field-based methods.

Spatial Twist Continuum (STC)[39] is an important tool in the whisker weaving method. STC is the dual representation of the quadrilateral/hex mesh. As one of the STC concepts, a sheet can be regarded as a general surface, which represents a layer of hex elements that share faces pairwise. The dual of a hex mesh is made of sheets. The curve, as the intersection of two sheets, is called a chord. A chord is dual to a continual chain of hexes connected with each other by their opposite faces. The arrangement of chords in a sheet is the dual of a quadrilateral mesh. If the process of connecting the sheets one by one is completed, then the whole arrangement of sheets is achieved. The intersection of a sheet with the geometric surface is a closed curve called a loop, representing the dual of a cycle of boundary surface quadrilaterals that share opposite edges pairwise.

The frame field is the key tool in the frame field-based method. It is defined in a tetrahedral mesh and can be considered as three vector fields that are continuous up to a simultaneous 3D rotation of all three fields consisting of *π*/2 rotations around the orthonormal axes. There are 3 vectors in every tetrahedral, and they define the value of the frame field of any point in this tetrahedral. The frame field is a guideline to drive meshing algorithms by providing geometric orientations inside the volume and on the boundary.

The frame field is used to guide the mesh generation. A chord represents the direction of the hex mesh. Accordingly, it inspires us to combine them to propose a new method for hex mesh generation, i.e., to use the frame field to guide the chord formation. It can consider the global information and use the frame field directly rather than parameterization. We introduce constrained quadrilateralization (CQ) for the first time to combine the STC with the frame field. Each boundary face is a part of two loops and is the starting point of a chord. The ending point of the chord is also part of the two loops noted above. Before weaving a sheet from a loop by chords, the two points of the loop that will be connected by a chord are determined. Every two points are constrained in this manner. Therefore, the CQ problem, as a sub-problem of hex mesh generation, is introduced. Solving the CQ problem which means that weaving a sheet from a loop is guided by the frame field.

We developed a hex mesh generation method via CQ. This method first divides the solid into two smaller sub-solids with a sheet and then considers the sub-solids recursively. The method to obtain chord arrangement of the sheet is to solve the CQ problem.

The remaining sections of the paper are as follows: Section 2 introduces some of the terminologies involved in this paper and the logical frame in our hex mesh generation method. Section 3 describes the steps of solving the CQ problem. The implementation of our method is illustrated in Section 4. Section 5 describes and analyzes the test results and comparisons of our method. Sections 6 and 7 give the discussion of the results and the conclusion, respectively.

## 2. Terminology and logical frame

We explained above that CQ is a sub-problem of the hex mesh generation. Solving the CQ problem is a sufficient condition for solving the problem of hex mesh generation. We build a logical framework from CQ to hex mesh generation. Some terms and definitions are first introduced before illustrating the framework.

### 2.1 Terminology

**Chord in 2D** is the dual of a continual chain of quadrilaterals connected with each other by opposite edges.

**Chord constraint** considered here is the requirement that any certain pair of boundary edges should be connected to each other by a chord in the final quadrilateral mesh. In other words, any edge has one and only one other edge that is in the same chord as it is in.

**CQ** is a type of quadrilateral mesh generation under the chord constraint. The starting point of the CQ is a polygon, which is composed of a sequence of edges connected from the beginning to the end. These edges should all satisfy the chord constraint.

**Hex mesh generation** refers to the filling of the interior of a solid model with hexes, starting with a quadrilateral mesh of the boundary surface of the solid model.

**Whisker** is an end of chord that is not completed, and its dual is a face (3D) or an edge (2D). The whisker is the basic object of the following basic operations.

**Sheet** is the dual of a layer of hex elements that share topological parallel faces with each other.

**Chord in 3D** is the intersection of two sheets and is the dual of a chain of hex elements connected with each other by opposite faces.

### 2.2 Logical framework

An arrangement of chords in a sheet is created via intersections of the sheet with all other sheets. A loop represents a chain of quadrilaterals among a closed-surface quadrilateral mesh. There is a sheet through the geometric model for each loop. The loop is the boundary of the sheet. A loop in a certain way defines a sheet. The arrangement of sheets is the dual of a hex mesh and is a key focus of this study. First, the arrangement of chords in each sheet is obtained, and then the arrangement of sheets is obtained naturally. Finally, a primal hex mesh can be constructed using a “gift-wrapping” algorithm [40] from the arrangement of the sheets.

The arrangement of chords in a sheet is dual to a quadrilateral mesh; thus, to obtain an arrangement of chords is to obtain the quadrilateral mesh that is dual to the arrangement.

**Fig 1:**
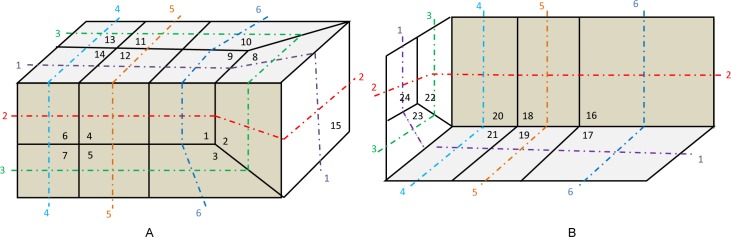
Surface mesh and loops of the “little corner”. (A) front, top and right faces; (B) back, bottom and left faces.

A quadrilateral on a loop is also on another loop if the loop is not self-intersecting. Therefore, two points which are connected with each other by a chord are constrained before obtaining the arrangement of chords in a sheet. The geometry and surface mesh for the example, referred to here as the “little corner” problem, are shown in Fig 1, where the two views show the front faces (Fig 1A) and a cutaway view of the back faces (Fig 1B). Faces and loops are also numbered for reference. Faces 12 and 19 are both on loop 1 and loop 5. Thus, these faces are the two ending points of the chord, which is the intersection of the sheet bounded by loop 1 and the sheet bounded by loop 5; that is, these two faces are under the chord constraint. Similarly, other faces are under their own chord constraints. Therefore, CQ is proposed as a sub-problem of hex mesh generation.

After a sheet is woven, a layer of hexes is formed. This layer of hexes divides the solid into two smaller sub-solids. The surfaces of these two sub-solids have been already meshed by quadrilaterals. Naturally, the sub-solids can be divided recursively until all sub-solids are empty.

Therefore, the framework involves the following: divide the current solid into two sub-solids by weaving a loop of the current solid and then address the sub-solids recursively.

### 2.3 Overview of the hex mesh generation method

**Fig 2:**
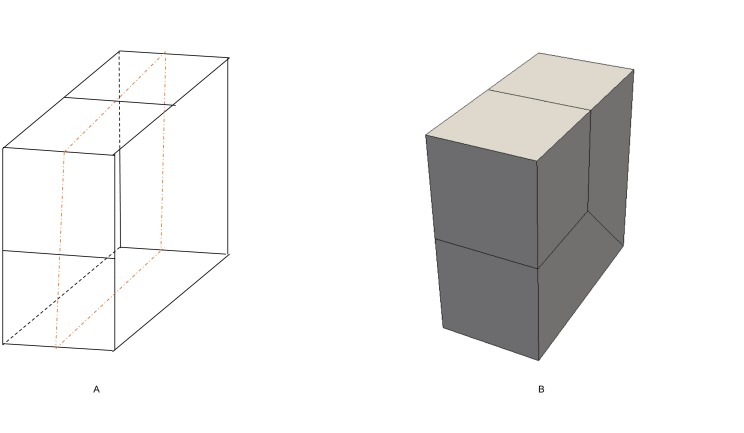
Loop 5 and the layer obtained by extending the loop 5. (A) loop 5; (B) the layer.

**Fig 3:**
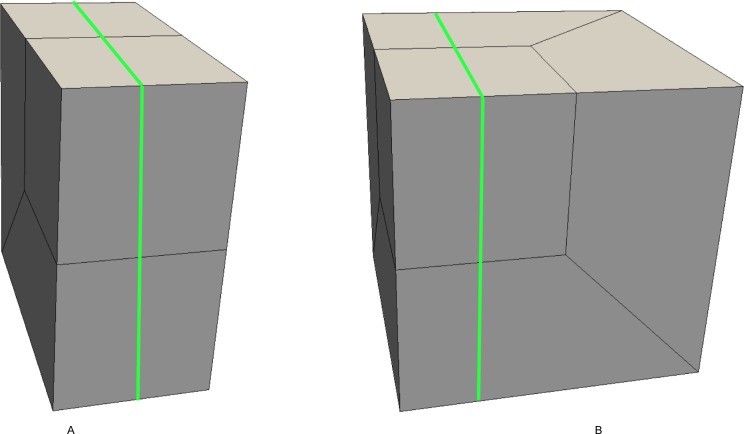
The two sub-solids after dividing by the layer. (A) the left sub-solid; (B) the right sub-solid.

**Fig 4:**
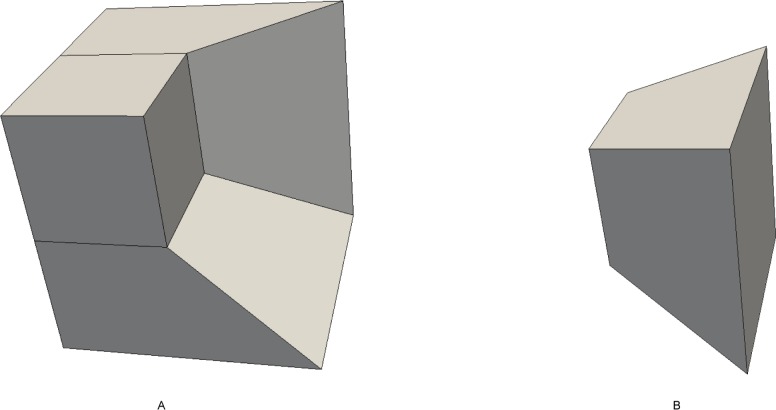
Address the right sub-solid recursively. (A) the layer; (B) the smaller sub-solid.

**Fig 5:**
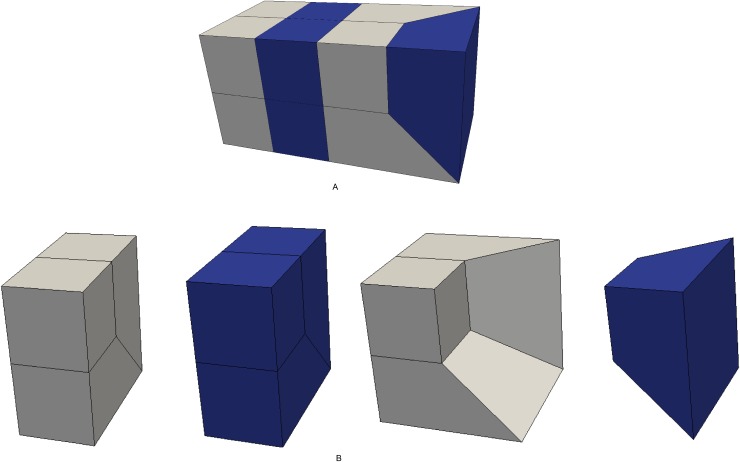
The hex mesh of the “little corner”. (A) the hex mesh; (B) open view.

We develop a method for hex mesh generation based on the logical framework mentioned in the above subsection. The whole algorithm of this method is briefly outlined in the followings steps:

1. Search the bisecting loop. Every loop divides the surface into two parts. The bisecting loop is the loop whose difference between one part’s number of quadrilateral and those of the other part is the smallest. Loops 1, 2, 3 and 5 can all be the bisecting loop in Fig 1. We select loop 5(see Fig 2A) as the bisecting loop here.
2. Obtain the arrangement of chords in the sheet bounded by loop 5. The process of the solution is detailed in Section 3. Next, extend the resulting quadrilateral mesh to a layer of hexes, as illustrated in Fig 2B.
3. Evaluate the layer and the two remaining parts. This layer divides the solid into two smaller sub-solids, as shown in Fig 3.
4. Address these two sub-solids. First, address the left sub-solid and select the loop with the green line as the bisecting loop (see in Fig 3A). After extending the resulting quadrilateral mesh, we find that both sub-solids of the left sub-solid are empty. Therefore, the processing of the left sub-solid is completed.
5. Next, address the right sub-solid. Select the loop with a green line in Fig 3B as the bisecting loop and obtain the layer of hexes (see Fig 4A). One of the sub-solids is empty, and the other one is shown in Fig 4B; recursively address this sub-solid. The further sub-solids are both empty. Thus, the recursion is ended.
6. Assemble all the layers during the process and the hex mesh of the original solid, as illustrated in Fig 5.

### 2.4 Multiple loops with a single sheet condition

**Fig 6:**
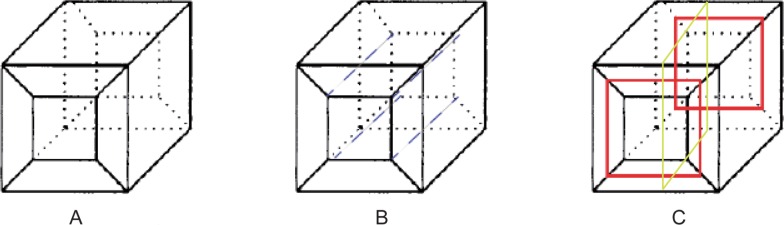
Two loops with a single sheet. (A) the surface mesh; (B) the solid mesh; (C) two loops with one sheet.

A problem should be discussed here. A surface (sheet) whose genus is more than 0 intersects the solid surface with more than one loop, while a surface (sheet) whose genus is 0 intersects the solid surface with only one loop. A quadrilateral surface mesh of a solid is illustrated in Fig 6A, and the solid is meshed into a hex mesh with five hexes, as illustrated in Fig 6B. The two loops with red lines shown in Fig 6C are at the same sheet. In this condition, the loop that can divide these two loops, such as the yellow one, is preferred to be selected as the bisecting loop. Next, revise the chord index of the bisecting loop guided by the frame field.

## 3. CQ algorithm

Before detailing the steps of the hex mesh generation method, we should first present the algorithm for CQ, which is the key step of the hex meshing method. The block diagram of this algorithm is as follows.

**Algorithm 1: CQ algorithm**

1.    INPUT boundary of domain, chord constraint and frame field

2.    CREATE initial dual based on the input

3.    DEFINE front and waiting whisker pair (WWP)

4.    LET S be the set of WWP

5.    LET M be the set of nodes and elements of the resulting quadrilateral mesh

6.    INPUT S, ADVANCE front using basic operations and OBTAIN M

7.    OPTIMIZE M and OUTPUT M

**Fig 7:**
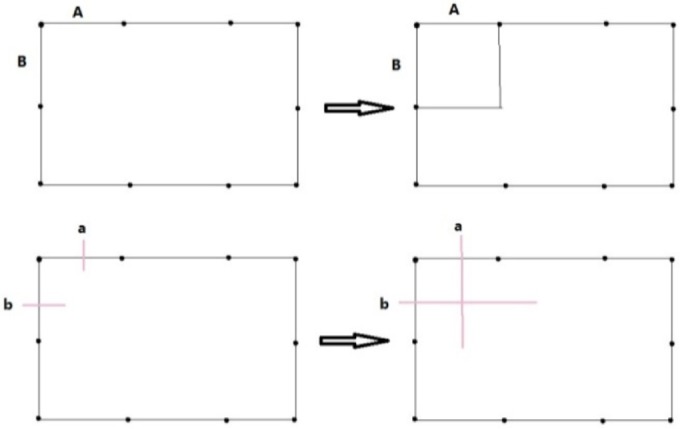
Two representations of cross operation.

**Fig 8:**
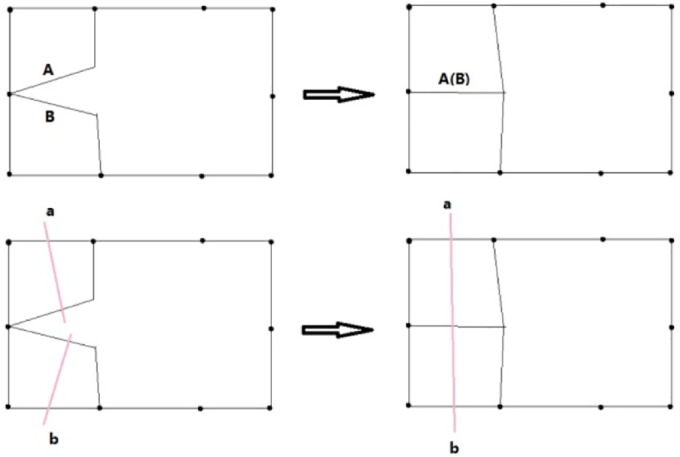
Two representations of the join operation.

**Fig 9:**
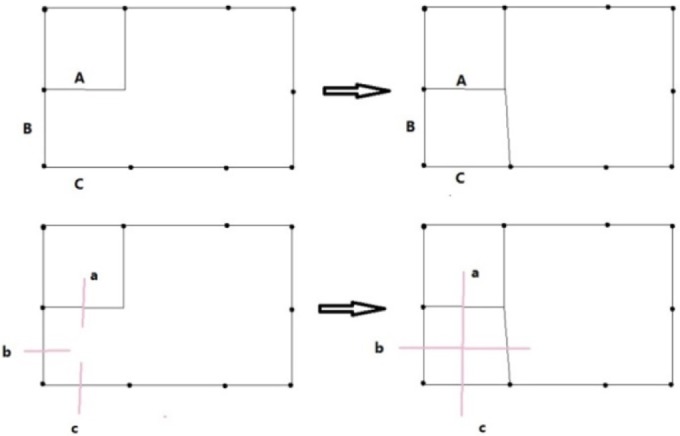
Two representations of CEJ.

This algorithm is outlined in the following steps:

1. **The Initial Dual**. The input of the algorithm is the information of the nodes and edges of the boundary polygon, the chord index of every edge and the normal of the nodes of the boundary polygon. Every edge has one and only one corresponding edge, whose chord index is the same as itself. Its dual is defined as a whisker. Thus, the number of chords is half the number of edges. Every two adjacent whiskers form a whisker pair (WP). A WP is a dual of the node shared by the two edges, which are the dual of the two whiskers. The WP is also an uncompleted 2-cell. The outer valence of the node is defined as the segment number. The normal is used for 3D geometry computation. Another input is the frame field.
2. **Front Definition**. The initial front is defined as the boundary of the domain. Every whisker is related to an edge. In the point of the dual, the front is a series of whiskers. The remaining ordered whiskers will be intersected, and the new front will be formed during processing. Whiskers a and b are related to the edges, as illustrated in Fig 7.
3. **Waiting Whisker Pair Definition.** We define a waiting whisker pair (WWP) to be a WP satisfying some conditions detailed below. Waiting indicates that the WP is to be chosen to be operated in the next step. WWP is an important concept in this algorithm. There are two kinds of WWP: initial WWP and general WWP.
  The initial WWP is defined from the initial WPs of the inputting boundary. There are many WPs in the inputting boundary, but only some of them can be operated by basic operations because the final mesh will be invalid after the others are operated. Below are the requirements that an initial WWP must satisfy.
  • Chord index. The chord index of the WWP’s two whiskers must be different. If they are in the same chord, then they must have been already joined. "Join" denotes edge moving. However, the edges that are dual to them cannot be moved because they are on the boundary.
  • Segment number. The initial WP whose segment number is two will form a poorly shaped quadrilateral; therefore, the WWP’s segment number must be larger than two.
  The general WWP is defined from general WPs during processing. The number of WPs decreases as the front advances, and some related WPs are changed after a basic operation. Therefore, we find and check the changed WPs to determine whether they are WWPs. A general WWP must meet the following requirements.
  • Segment number. The WWP segment number must be greater than one because a WWP whose segment number is one will form a doublet after a basic operation. Doublets must be forbidden in the final mesh.
  • Intersect state. A whisker intersects increasing numbers of whiskers during the front processing. We define a matrix to record the number of intersections of each two chords. Each of the two parallel chords intersection times must not be greater than two, and each of the two non-parallel chords intersection times must not be greater than one. We consider two chords to be parallel if the starting and ending points of a chord are on the same side of the other chord. Only if this requirement is met can the final mesh be simple.
4. **Basic Operations**. Basic operations define how to operate a WP. Once a WP is operated, the front advances a step. There are three basic operations shown in Figs 7, 8 and 9.
  • **Cross**. If two whiskers of a WP are not on the same chord (i.e., their chord indices are different), they can cross with each other. In Fig 7, whiskers a and b at the bottom-left have different chord indices. Edges A and B, which are dual to whiskers a and b at the top-left, respectively, are in different chords. A quadrilateral at the top-right is formed after cross. At the same time, a crossing (the dual of quadrilateral) at the bottom-right is formed.
  • **Join**. If two whiskers of a WP are on the same chord, they can be merged, and these two uncompleted chords (whiskers) become completed. In Fig 8, whiskers a and b at the bottom-left have the same chord index. Edges A and B, which are dual to whiskers a and b at the top-left, respectively, are in the same chord. Edges A and B are merged into one edge at the top-right after performing the join operation. At the same time, whiskers a and b at the bottom-right are joined into a chord and the chord to which they belong is completed.
  • **CEJ**. The two adjacent WPs have 3 whiskers because a whisker is shared by the two WPs. If the first and the third of these three whiskers are on the same chord, then the first and second whisker can cross each other; then, the first and the third whisker can be merged. This operation is called cross-embedded join (CEJ), which is illustrated in Fig 9. Whiskers a and c shown at the bottom-left have the same chord index, while whisker b differs from them. Edges A and C shown at the top-left are on the same chord. A quadrilateral is formed, and edges A and C shown at the top-right are merged after CEJ. Moreover, a crossing is formed and whiskers a and c shown at the bottom-right are joined. Because a cross and a join is used in CEJ, we conclude that CEJ = Cross + Join, in some sense.
5. **Front Processing**. WPs are operated in the following order. The closer to the frame field the whiskers of the WP are, the more preferential the WP is to be operated. Once a WP is operated using one of three basic operations, the front advances (a quadrilateral is formed, two edges of front are merged or both) and becomes a new front. The remaining WPs are updated and become new WPs. New WPs will be processed recursively.
6. **Special Process**. Front processing continues until meeting the condition that the remaining void can be subdivided by quadrilaterals without adding nodes. Next, a special process is performed until the completed quadrilateral mesh is obtained.
7. **Optimization of Patch**. The quality of the obtained quadrilateral mesh is poor in some places. Patches are created around irregular nodes, and then they are reconnected to optimize the valence.

**Fig 10:**
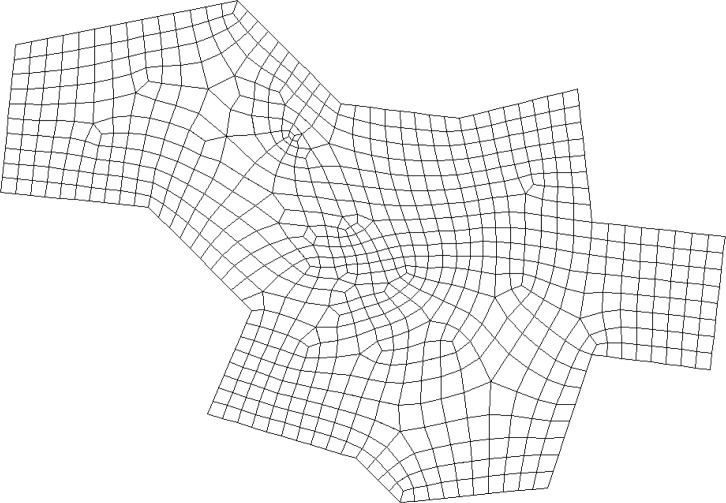
An example of CQ.

CQ is guided by the frame field so that the resulting quadrilateral mesh is consistent with the frame field. Note that we use the frame field as guide here. We can also use non-uniform rational B-splines (NURBS) as a guide to generate a quadrilateral mesh as shown in Fig 10 under the chord constraint. In this example, the chord constraints are set and the two edges with the same chord index are used to construct the NURBS to guide the front advancing.

## 4. The implementation of the hex mesh generation

The divide-and-conquer strategy is used in the hex mesh generation method. The following is a more detailed discussion of this method addressing some of the more important implementation issues.

### 4.1 Input the quadrilateral mesh of the solid surface and the frame field

One of the inputs of this method is the frame field that is defined in a tetrahedral mesh. The other input is the surface quadrilateral mesh, which conforms to the frame field. The frame field is used to guide CQ.

### 4.2 Construct the solid box

Here, the quadrilateral mesh of solid surface is called a solid box. The solid box is constructed based on the input data of the surface quadrilateral mesh.

### 4.3 Address the solid box

Addressing the solid box is an important step in our method. The sub-steps of addressing the solid box are as follows:

1 **Analyze the mesh of the solid box**. Analyze the configuration of the loops based on inputting elements and nodes. Revise the chord index of every quadrilateral and revise quadrilaterals on each loop. The chord in a closed-surface quadrilateral mesh is the loop in 3D.
2 **Obtain the bisecting loop.** We must consider geometric information in selecting the bisecting loop. The selecting process refers to Algorithm2. Count the number of quadrilaterals in the two sides of the loop. One side is labeled as the positive side, whereas the other side is labeled as the negative side. Next, detect the crease edges of the loop. A crease edge is an edge whose two corresponding faces form a sharp dihedral angle. Subsequently, select the loop whose edges are all creased or none of whose edges are creased and whose difference between one part’s number of quadrilaterals and the other part’s is the smallest.

**Algorithm 2: Obtain the bisecting loop**

1.    SET the minimum difference between two sides of the loop *FaceCount*_*min*_ = ∞ and the id of minimum loop as *min*

2.    SET the flag of positive side as *PosFlag* and the flag of negative side as *NegFlag*

3.    FOR each loop *i*

4.        SET the number of crease edges of positive side *PosCount* = 0 and the number of crease edges of negative side *NegCount* = 0

5.        FOR each quadrilateral on loop*i*

6.    IF (positive edge is crease)

7.    *PosCount*++

8.            IF (negative edge is crease)

9.    *NegCount*++

10.            Count the numbers of the quadrilaterals of two sides

11.            Record the difference between the two sides *FaceCount*

12.        END FOR

13.        IF (*FaceCount*_*min*_>*FaceCount* and (*PosCount* = = 0 or all) and (*NegCount* = = 0 or all))

14.            *FaceCount*_*min*_ = *FaceCount*

15.            *min* = *i*

16.      END FOR

17.      AS FOR loop *min*

18.      IF (*PosCount* = = 0)

19.        *PosFlag* = 0

20.      ELSE

21.        *PosFlag* = 1

22.      IF (*NegCount* = = 0)

23.        *NegFlag* = 0

24.      ELSE

25.        *NegFlag* = 1

Selecting the loop with minimum difference aims at dividing the solid box into two as equal as possible parts and can speed the process. Note that the solution space tree is a binary tree, and the time complexity is related to height of the tree. Obviously, the height of balanced binary tree is the minimum. Therefore, it is necessary to select the loop with minimum difference.

**Fig 11:**
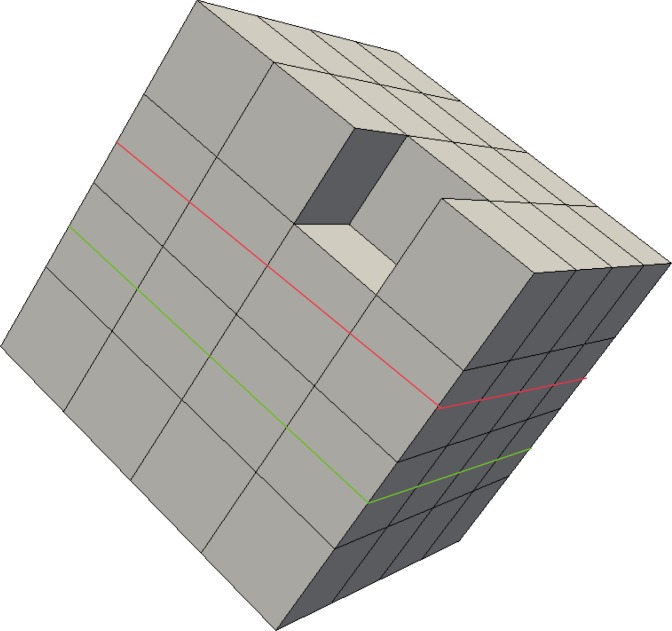
The selection of the bisecting loop.

The loop with all crease edges or no crease edges is selected because, in this condition, it is easy to divide the sub-solid box further. If the loop has not only crease edges but also general edges, the sub-solid box is difficult to address. For example, in Fig 11, the red loop has only one crease edge, and the shape of positive sub-solid is unusual, with one empty part and another non-empty part. Addressing this sub-solid is very time consuming because it requires a surface-surface intersection. In this condition, we should select the green loop as the bisecting-loop.

Note that if the multiple loops with single sheet condition exits, then the loop that can divide these loops should be selected first.

**Fig 12:**
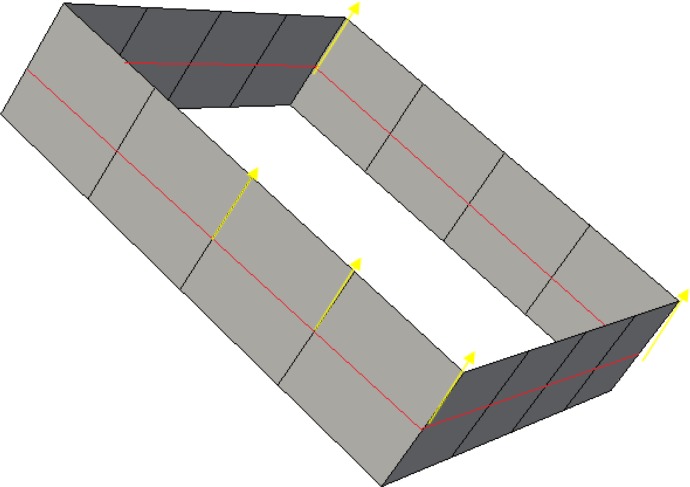
The input data of CQ mesh generation.

**Fig 13:**
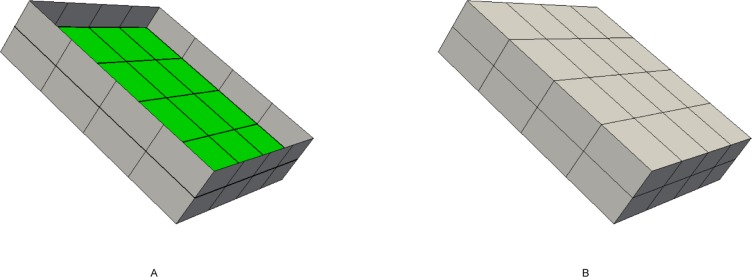
Extend the quadrilateral mesh to the hex layer. (A) the output of CQ mesh generation; (B) the layer of hexes.

3 **Construct the boundary edges, chord constraint and the normal of boundary nodes**. The loop obtained in the last sub-step is a continual chain of quadrilaterals connected with each other by opposite edges and is named the loop band. Choose the midpoint of the inner edges of the loop band and connect them to form a polygon, such as the red polygon shown in Fig 12. Choose the other chord index as the chord constraint of the polygon. Choose the inner edges vector as the normal of boundary nodes, as indicated by the yellow arrows shown in Fig 12. If the multiple loops with single sheet condition exits, then we should evaluate the chord constraint. Move from the coordinate of every whisker along the direction of the frame field until encountering another whisker. If these two whiskers have different chord indices, then evaluate the other two whiskers whose chord indices are the same as theirs. If these whiskers meet each other, exchange their chord indices. The chord indices of all whiskers can be revised in this manner.
4 **Solve the problem of CQ**. Input the polygon, chord constraints, normals and frame field and then solve the CQ problem (see Section 3). A quadrilateral mesh admiring the chord-constraint and frame field will be output.
5 **Extend the quadrilateral mesh to a layer of hexes.** This sub-step is conducted to advance the quadrilateral mesh along the normal directions and the negative normal directions and to obtain two other quadrilateral meshes. The boundary nodes of these two quadrilateral meshes are the nodes on the side edges of the loop band. Inner nodes in quadrilateral meshes can be obtained by interpolation of boundary nodes. Next, connect the element of these two meshes to form hexes. A layer of hexes is formed as illustrated in Fig 13B. The green quadrilateral mesh in Fig 13A is the output of CQ.
6 Construct and address the sub-solid. Remove the layer of hexes from the original solid, and the shapes of the sub-solids are related to the property of the side edges of the loop band. The type of sub-solids can be defined by two bits. If *PosFlag* is 1, the positive sub-solid box is empty. If *NegFlag* is 1, the negative sub-solid box is empty. This relationship is detailed in Table 1. If the sub-solid is not empty, steps (1)-(6) will be repeated to address it.

**Table 1:** Descriptions of the four types of sub-solids.

type	positive	negative
0–0	Not empty	Not empty
1–0	Empty	Not empty
0–1	Not empty	Empty
1–1	Empty	Empty

### 4.4 Assemble and output hex mesh

When all the sub-solids are empty, the original solid is meshed completely. All of the hex layers are assembled into a hex mesh.

## 5. Experimental results and comparisons

We choose some examples to demonstrate the experimental results of our method. Our machine is a Xeon E5-2630 PC (PRECISION T5600 DELL, 2.3 GHz CPU and 64 GB RAM) with the Visual C++ 10 compiler. The quality of the hex element is measured by the value of the Jacobian matrix[41] labeled as *J*. *J* is in the range [-1, 1]. The higher *J* is, the more regular the hex is and the better the quality is. We choose the average value of *J* of all the elements labeled as *J*_*avg*_ and the minimum of *J* of all the elements labeled as *J*_*min*_ as the metric of the whole mesh quality. Because our method is guided by frame field, we use a field-guided method, such as FSC(frame field singularity correction) [8] and SRF(singularity-restricted field) [38] for comparison.

### 5.1 Comparison to the FSC method

**Fig 14:**
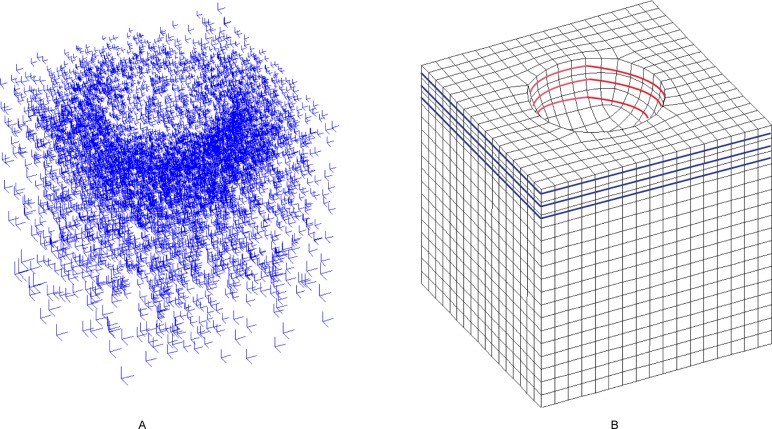
The input of example 1. (A) the frame field of example 1; (B) the quadrilateral mesh of example 1.

**Fig 15:**
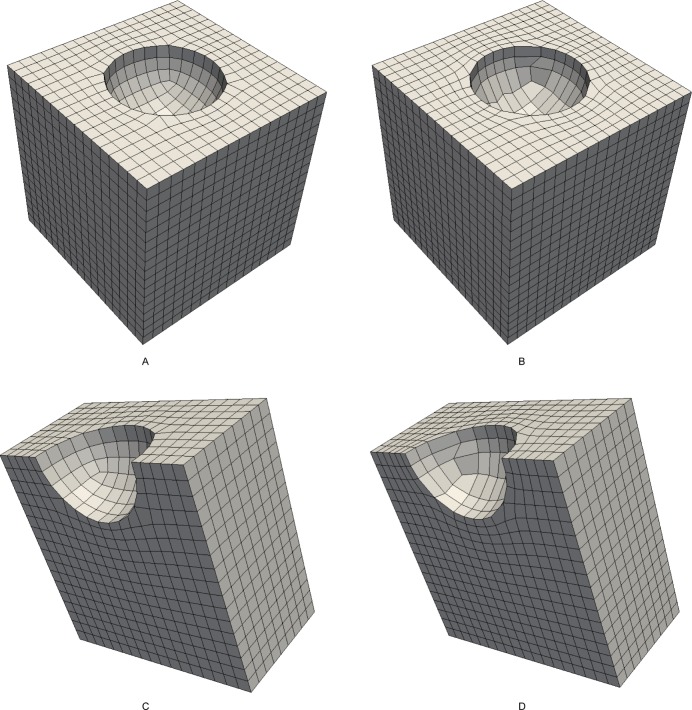
The comparison of meshes of example 1. (A) the outside view of our resulting mesh; (B) the outside view of FSC mesh; (C) the cutaway view of our resulting mesh; (D) the cutaway view of FSC mesh.

The model of example 1 is the remaining shape after a half-sphere has been subtracted from a cube. The input frame field and quadrilateral mesh are illustrated in Fig 14A and 14B, respectively. The resulting hex mesh is shown in Fig 15A and 15C. The mesh generated by the FSC method is shown in Fig 15B and 15D. Table 2 shows the result of example 1. The qualities of our mesh and the FSC mesh are both improved by Mesquite[42]. We can see that both the average quality and the minimum quality of mesh generated by our method are better than those generated by the FSC method. The quadrilaterals near the sphere in the former mesh are closer to square than the ones in the latter mesh. Both the numbers of the nodes and the elements of the latter mesh are greater than in the former mesh; thus, the quality of the latter mesh is theoretically better than the former. However, based on Table 2, the latter is worse than the former. We also find that the numbers of singular vertices in our mesh and FSC mesh are both 0, which means both meshes are ideal in terms of topology.

**Table 2:** Statistics for example 1.

Example 1	nodes	element	*J*_avg_	*J*_min_	singular vertices	time/s
our method	5 724	4 766	0.992	0.309	0	32.2
FSC	6 784	5 724	0.990	0.301	0	

There are two loops with the single sheet condition in example 1. The top three layers of the quadrilaterals correspond to six loops: three on the cube, marked in blue, and three on the sphere, marked in red. Every two loops correspond to one sheet. Our method can handle this condition.

### 5.2 Comparison to the SRF method

**Fig 16:**
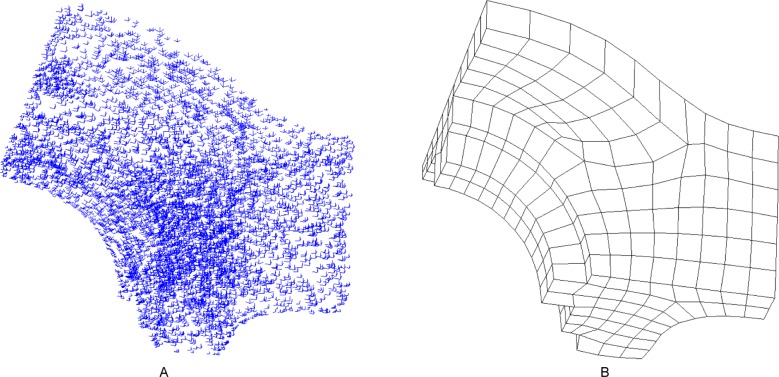
The input of example 2. (A) the frame field of example 2; (B) the quadrilateral mesh of example 2.

**Fig 17:**
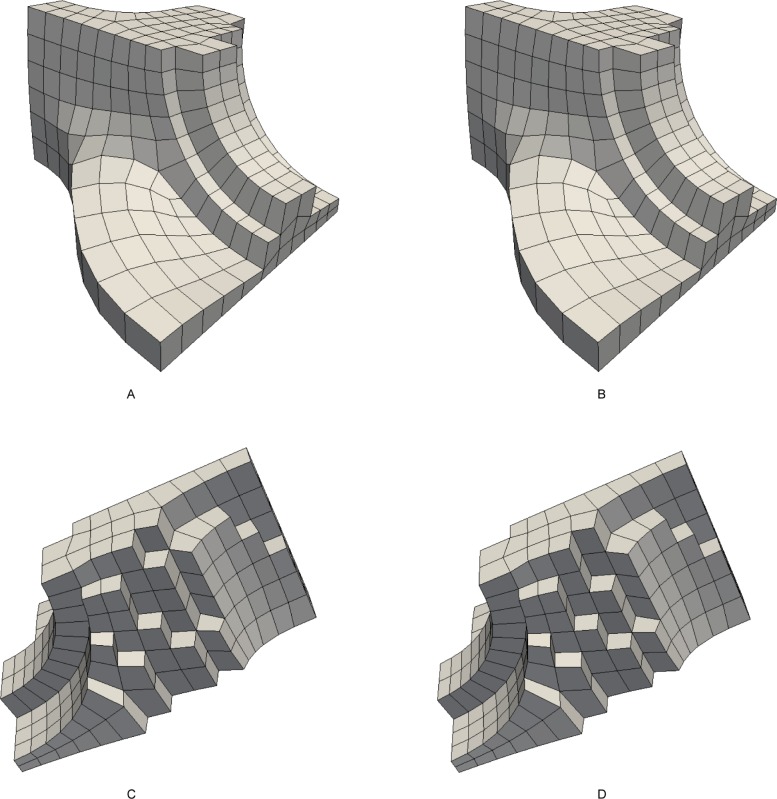
The comparison of meshes of example 2. (A) the outside view of our resulting mesh; (B) the outside view of SRF mesh; (C) the cutaway view of our resulting mesh; (D) the cutaway view of SRF mesh.

**Fig 18:**
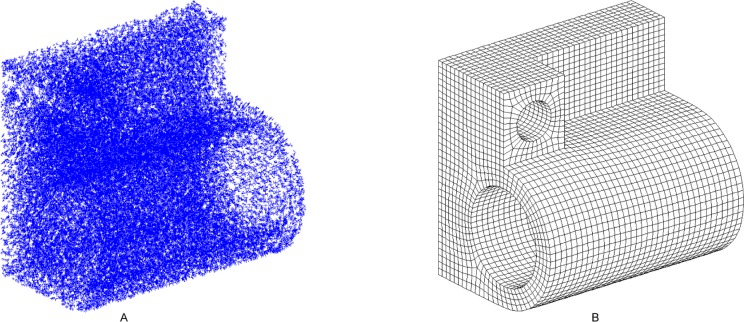
The input of example 3. (A) the frame field of example 3; (B) the quadrilateral mesh of example 3.

**Fig 19:**
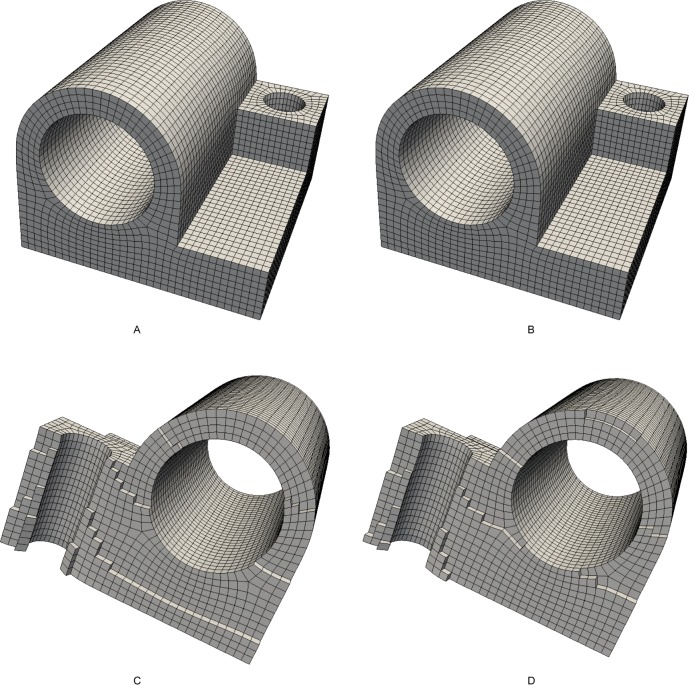
The comparison of meshes of example 3. (A) the outside view of our resulting mesh; (B) the outside view of SRF mesh; (C) the cutaway view of our resulting mesh; (D) the cutaway view of SRF mesh.

Example 2 and example 3 consider the models “fandisk” and “joint”, respectively. We compare the SRF method with our method. The input frame field and the quadrilateral mesh of example 2 are illustrated in Fig 16A and 16B, respectively. The resulting hex mesh is shown in Fig 17A and 17C. The mesh generated by the SRF method is shown in Fig 17B and 17D. Similarly, the input field and mesh of example 3 are illustrated in Fig 18, and the output hex mesh is shown in Fig 19. The statistics of the two examples are listed in Table 3. The qualities of our meshes are improved by Mesquite. The mesh files of SRF are downloaded from [38]. According to [38], the qualities of all hex meshes have been improved by Mesquite. There are few differences between the meshes generated by the two methods: the numbers of elements and nodes are the same, the average qualities are also the same, and only the worst quality of our method is slightly better than that of the SRF method. Therefore, the effect of our method is similar to that of the SRF method. The numbers of singular vertices in our meshes and the SRF meshes are equal.

**Table 3:** Statistics for examples 2 and 3.

		nodes	element	*J*_avg_	*J*_min_	singular vertices	time/s
Example 2	our method	614	357	0.936	0.614	10	2.8
	SRF	614	357	0.936	0.609	10	
Example 3	our method	21 654	17 784	0.984	0.737	130	105.6
	SRF	21 654	17 784	0.984	0.729	130	

There also exist multiple loops with single sheet conditions near cylinders in example 3. Our method can handle these conditions.

### 5.3 Analysis

The testing results show that our method working on the mesh level generates meshes with the same quality as the methods working on the field level. Our method uses the same field as that of the FSC method and SRF method. The only difference is how to use the field. The former forms mesh elements directly guided by the field. The latter first computes a volumetric parameterization guided by this field and then extracts a hex mesh to which the parameterization leads. These methods can all generate satisfactory meshes.

Note that we directly use the result files of the FSC method and the SRF method; thus, we cannot obtain the running time of these two methods. We still list the running time of our method for the three examples. We find that our method runs efficiently.

## 6. Discussions

The various kinds of methods introduced in the Introduction have similarities with our method. The comparisons between those methods and our method are as follows. The first kind of method is the parameterization-based methods. These methods differ from our method in how the mesh is obtained via the frame field. Our method directly drives the sheet generation by working on STC, while these methods generate volumetric parameterization. Thus, our method can potentially lead to easier and more robust implementations that are more parallelizable and less dependent on heavy numerical libraries. The second kind is developed whisker weaving methods. For example, references [17, 18, 20, 43] all form a layer of hexes using loop elimination. The difference is that these methods often eliminate a loop from the border of the solid, whereas our method is from the “center” of the solid. The mesh of the layer of hexes can be guaranteed to be consistent for the frame field’s guiding. The third kind is the method proposed by Calvo[44]; it directly finds a topological bisecting loop and then projects a connection of one of the sides to this middle layer. This process is too simple to obtain meshable sub-solids. Our method forms the hex layer with the guidance of the frame field so that all the sub-solids are meshable.

It should be noted that if the loops of the surface quadrilateral mesh are self-intersecting, our method will fail. In this condition, the loop will form a complicated shape such “8”, and the CQ algorithm cannot handle this kind of boundary. Because the surface quadrilateral mesh generation guided by frame field is very mature, however, the loops of the generated meshes are seldom self-intersecting. Therefore, our method does not consider the self-intersecting condition; this is also common in field-guided methods.

Our method depends upon the frame field: 1) boundary dependence, i.e., the inputting surface quadrilateral mesh depends on the frame field; 2) inner dependence, i.e., in advancing the front in CQ, the trend of whiskers depends on the field. All generated frame fields have some drawbacks; thus, our method can only handle finite scale models. Because it is difficult to obtain the field for large models, they cannot be meshed successfully by our method. These dependences are the drawbacks of our method. Developing our own frame field generation method can further deepen our understanding of the underlying frame field and can increase the control for the frame field, making it more useful. Therefore, developing the frame field technique is one of our future works.

This method is based on the divide-and-conquer strategy. The divide-and-conquer strategy is the basic principle of the parallel algorithm, making it suitable to perform parallel transformation, which is also future research area.

## 7. Conclusions and future work

We propose a hex mesh generation method via CQ. In this method, the original solid is divided into two smaller sub-solids, which can then be processed recursively. Each step of the division involves solving the CQ problem. We developed an algorithm to solve the CQ problem. Our method can potentially lead to easier and more robust implementations that are more parallelizable and less dependent on heavy numerical libraries. The experimental results show that our method produces similar results as the results of the current state-of-the-art mesh generation methods.

The frame field plays an important role in our method. Developing our own frame field generation method will be undertaken in our future works. The divide-and-conquer strategy used in our method is suitable for parallelization. Developing a parallel implementation of our method is also one of the studies to be performed in the future.

## Appendix

The spatial twist continuum (STC) that quantifies the global connectivity information of hexahedral meshes is a very important tool for our algorithm. We give a brief introduction of the STC definitions below.

**Fig 20:**
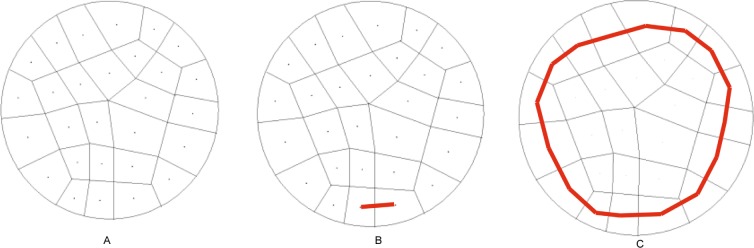
STC entities. (A) STC centroids; (B) a STC edge; (C) a 2D chord.

The 2D STC is a powerful extension of the dual representation of a quadrilateral mesh. Fig 20 shows some STC dual entities of a simple quadrilateral mesh. In Fig 20(A), we pick one point inside every quadrilateral as the dual of the quadrilateral, which is called the STC centroid. The corresponding centroids of two quadrilaterals sharing a mesh edge are connected to each other by a STC edge. Every STC edge corresponds to a mesh edge. A dual edge is shown in Fig 20(B). The opposite edges of a quadrilateral are topologically parallel. The curve made of STC edges corresponding to a group of topologically parallel mesh edges is called a STC chord, as illustrated in Fig 20(C).

**Fig 21:**
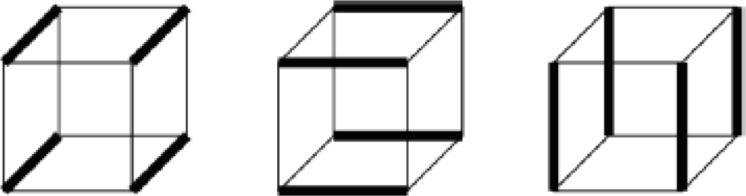
3 groups of topologically parallel edges of a hex.

**Fig 22:**
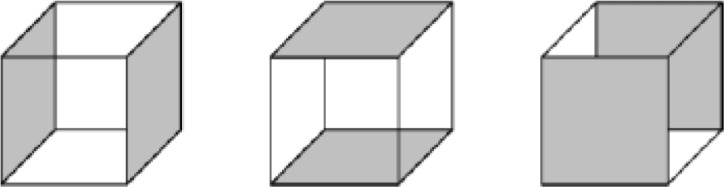
3 groups of topologically parallel faces of a hex.

**Fig 23:**
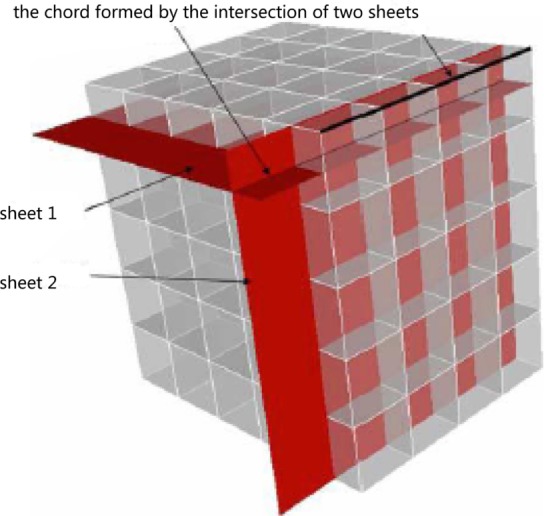
A chord and two sheets of a hex mesh.

Similarly, the 3D STC of a hex mesh is the extension of the 2D case. In the 3D case, in addition to the topologically parallel edges (see Fig 21), we also introduce the topologically parallel faces, i.e., the opposite faces of a hex, as shown in Fig 22. Therefore, we can define the 3D STC centroids, edges and cells in the same way as the 2D cases, and a 3D chord represents a continual chain of hexes connected with each other by their opposite faces. However, the most important concepts that differ from the 2D cases are the 2-cell and the sheet. A 2-cell is constructed by connecting the STC centroids around a hex mesh edge. Thus, an edge in the hex mesh corresponds to a 2-cell. Extending a 2-cell and bisecting all the topologically parallel edges of the relating edge results in a surface called a sheet. A sheet represents a layer of hexahedral elements that share faces pairwise. Fig 23 shows a chord as the intersection of two sheets.

**Fig 24:**
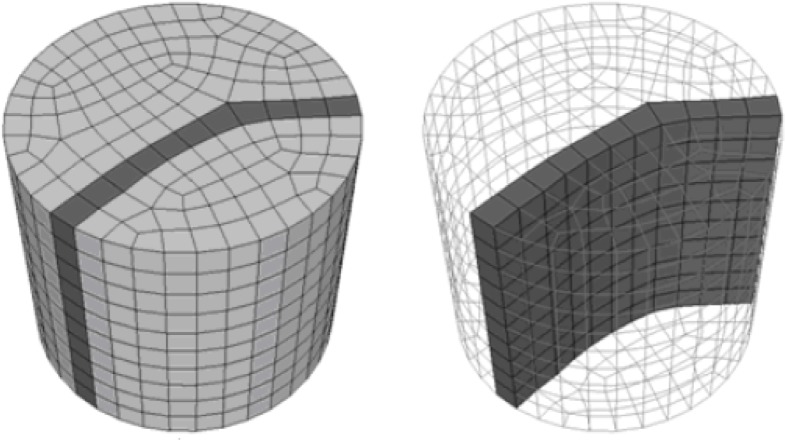
A surface loop corresponding to a sheet.

The intersection of a sheet with the geometric surface is a closed curve called a loop, representing the dual of a cycle of surface quadrilaterals that pairwise share topologically parallel edges. Each boundary face is part of two loops and is the starting point of a chord. As shown in Fig 24, a surface loop can be regarded as the intersection of a sheet with the boundary surface. In other words, one or more surface loops correspond to a sheet inside the hex mesh.

## Supporting information

S1 File The mesh file of example 1 generated by our method. For visualizing this mesh, please use free Software ParaView (http://www.paraview.org)
(VTK)

S2 File The mesh file of example 2 generated by our method. For visualizing this mesh, please use free Software ParaView (http://www.paraview.org)
(VTK)

S3 File The mesh file of example 3 generated by our method. For visualizing this mesh, please use free Software ParaView (http://www.paraview.org)
(VTK)
